# 1,4-Bis(3-chloro­pyrazin-2-yl­oxy)benzene

**DOI:** 10.1107/S1600536813007824

**Published:** 2013-03-28

**Authors:** Thothadri Srinivasan, Venkatesan Kalpana, Perumal Rajakumar, Devadasan Velmurugan

**Affiliations:** a Centre of Advanced Study in Crystallography and Biophysics, University of Madras, Guindy Campus, Chennai 600 025, India; b Department of Organic Chemistry, University of Madras, Guindy Campus, Chennai 600 025, India

## Abstract

In the title compound, C_14_H_8_Cl_2_N_4_O_2_, the pyrazine rings are orthogonal to the benzene ring, making dihedral angles of 88.42 (8) and 89.22 (8)°. The Cl atoms attached to the pyrazine rings deviate by −0.0597 (5) and 0.0009 (5) Å from the ring plane. The crystal structure features C—H⋯N hydrogen bonds.

## Related literature

For applications of the pyrazine ring system in drug development, see: Du *et al.* (2009 ▶); Dubinina *et al.* (2006 ▶); Ellsworth *et al.* (2007 ▶); Mukaiyama *et al.* (2007 ▶). For a related structure, see: Nasir *et al.* (2010 ▶).
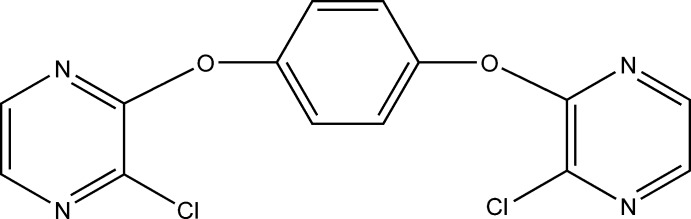


## Experimental

### 

#### Crystal data

C_14_H_8_Cl_2_N_4_O_2_
*M* *_r_* = 335.14
Monoclinic, 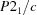

*a* = 11.083 (2) Å
*b* = 10.0452 (17) Å
*c* = 12.846 (2) Å
β = 105.681 (6)°
*V* = 1376.9 (4) Å^3^
*Z* = 4
Mo *K*α radiation
μ = 0.48 mm^−1^
*T* = 293 K
0.30 × 0.25 × 0.20 mm

#### Data collection

Bruker SMART APEXII area-detector diffractometer
Absorption correction: multi-scan (*SADABS*; Bruker, 2008 ▶) *T*_min_ = 0.869, *T*_max_ = 0.909
12550 measured reflections
3461 independent reflections
2835 reflections with *I* > 2σ(*I*)
*R*_int_ = 0.023

#### Refinement

*R*[*F* ^2^ > 2σ(*F* ^2^)] = 0.032
*wR*(*F* ^2^) = 0.092
*S* = 1.04
3461 reflections
199 parameters
H-atom parameters constrained
Δρ_max_ = 0.32 e Å^−3^
Δρ_min_ = −0.26 e Å^−3^

### 

Data collection: *APEX2* (Bruker, 2008 ▶); cell refinement: *SAINT* (Bruker, 2008 ▶); data reduction: *SAINT*; program(s) used to solve structure: *SHELXS97* (Sheldrick, 2008 ▶); program(s) used to refine structure: *SHELXL97* (Sheldrick, 2008 ▶); molecular graphics: *ORTEP-3 for Windows* (Farrugia, 2012 ▶); software used to prepare material for publication: *SHELXL97* and *PLATON* (Spek, 2009 ▶).

## Supplementary Material

Crystal structure: contains datablock(s) global, I. DOI: 10.1107/S1600536813007824/pv2624sup1.cif

Structure factors: contains datablock(s) I. DOI: 10.1107/S1600536813007824/pv2624Isup2.hkl

Supplementary material file. DOI: 10.1107/S1600536813007824/pv2624Isup3.cml

Additional supplementary materials:  crystallographic information; 3D view; checkCIF report

## Figures and Tables

**Table 1:** Hydrogen-bond geometry (Å, °)

*D*—H⋯*A*	*D*—H	H⋯*A*	*D*⋯*A*	*D*—H⋯*A*
C13—H13⋯N3^i^	0.93	2.60	3.480 (2)	159

Symmetry code: (i) 
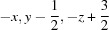
.

**Fig. 1.**
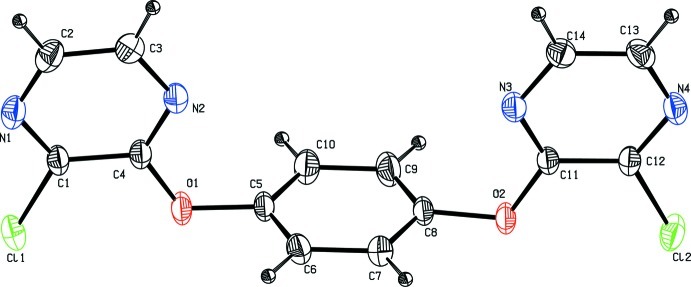
The molecular structure of the title compound, showing displacement ellipsoids drawn at the 30% probability level. H atoms are presented as small spheres of arbitrary radius.

**Fig. 2.**
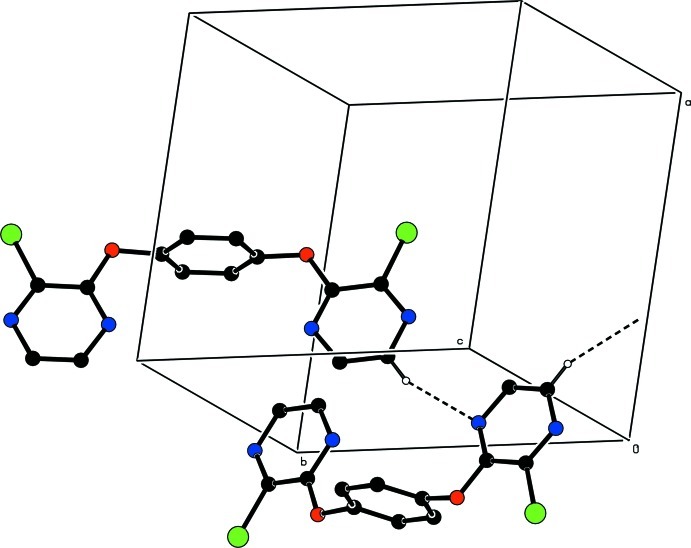
The crystal packing of the title compound viewed down c axis. H-atoms not involved in H-bonds have been excluded for clarity.

## supplementary crystallographic information

### Comment

The pyrazine ring is a useful structural unit in medicinal chemistry and has found broad applications in drug development and can be used as antiproliferative agent (Dubinina *et al.*, 2006), potent CXCR3 antagonist (Du *et al.*, 2009), CB1 antagonist (Ellsworth *et al.*, 2007) and c-Src inhibitor (Mukaiyama *et al.*, 2007). In view of different applications of this class of compounds, we have undertaken the single-crystal structure determination of the title compound.

The bond distances and angles in the title compound (Fig. 1) agree very well with the corresponding bond distances and angles reported in a closely related compound (Nasir *et al.*, 2010). In the titled compound, the pyrazine ring (N1/N2/C1-C4) makes a dihedral angle of 88.42 (8)° with the benzene ring (C5-C10), which shows that these are orthogonal to each other. The other pyrazine ring (N3/N4/C11-C14) makes a dihedral angle of 89.22 (8)° with the bezene ring, which also shows that these are also orthogonal to each other. The dihedral angle between the two pyrazine rings is 3.18 (7)°. The chlorine atoms Cl1 and Cl2 attached with the pyrazine rings deviate by -0.0597 (5) and 0.0009 (5)Å. The crystal packing is stabilised by intermolecular C–H···N hydrogen bonds (Tab. 1 & Fig. 2).

### Experimental

To a stirred solution of Cs_2_CO_3_/K_2_CO_3_ (22 mmol) in CH_3_CN (50 mL), dihydroxybenzenes (10 mmol) was added and stirred for 5 min. 2,3-Dichloropyrazine (20 mmol) in CH_3_CN (100 mL) was added dropwise to the above reaction mixture and stirring was allowed at refluxing condition for 12 h. After the reaction was complete, the reaction mixture was allowed to attain room temperature and then evaporated to dryness. The residue obtained was extracted with CH_2_Cl_2_ (3 x 100 mL), washed with water (3 x 100 mL), brine and then dried over Na_2_SO_4_. Evaporation of the organic layer gave a residue, which on purification using column chromatography with hexane/CHCl_3_ (1:1) as an eluent gave the corresponding compound. Single crystals suitable for X-ray diffraction were obtained by slow evaporation of a solution of the title compound in hexane at room temperature.

### Refinement

The hydrogen atoms were placed in calculated positions with C—H = 0.93 Å, refined in the riding model with fixed isotropic displacement parameters: *U*_iso_(H) = 1.2*U*_eq_(C).

### Crystal data

**Table:**

C_14_H_8_Cl_2_N_4_O_2_	*F*(000) = 680
*M**_r_* = 335.14	*D*_x_ = 1.617 Mg m^−^^3^
Monoclinic, *P*2_1_/*c*	Mo *K*α radiation, λ = 0.71073 Å
Hall symbol: -P 2ybc	Cell parameters from 3461 reflections
*a* = 11.083 (2) Å	θ = 1.9–28.5°
*b* = 10.0452 (17) Å	µ = 0.48 mm^−^^1^
*c* = 12.846 (2) Å	*T* = 293 K
β = 105.681 (6)°	Block, colourless
*V* = 1376.9 (4) Å^3^	0.30 × 0.25 × 0.20 mm
*Z* = 4	

### Data collection

**Table:**

Bruker SMART APEXII area-detector diffractometer	3461 independent reflections
Radiation source: fine-focus sealed tube	2835 reflections with *I* > 2σ(*I*)
Graphite monochromator	*R*_int_ = 0.023
ω and φ scans	θ_max_ = 28.5°, θ_min_ = 1.9°
Absorption correction: multi-scan (*SADABS*; Bruker, 2008)	*h* = −14→14
*T*_min_ = 0.869, *T*_max_ = 0.909	*k* = −13→12
12550 measured reflections	*l* = −17→17

### Refinement

**Table:**

Refinement on *F*^2^	Primary atom site location: structure-invariant direct methods
Least-squares matrix: full	Secondary atom site location: difference Fourier map
*R*[*F*^2^ > 2σ(*F*^2^)] = 0.032	Hydrogen site location: inferred from neighbouring sites
*wR*(*F*^2^) = 0.092	H-atom parameters constrained
*S* = 1.04	*w* = 1/[σ^2^(*F*_o_^2^) + (0.0434*P*)^2^ + 0.4056*P*] where *P* = (*F*_o_^2^ + 2*F*_c_^2^)/3
3461 reflections	(Δ/σ)_max_ = 0.001
199 parameters	Δρ_max_ = 0.32 e Å^−^^3^
0 restraints	Δρ_min_ = −0.26 e Å^−^^3^

### Special details

**Table:**

Geometry. All esds (except the esd in the dihedral angle between two l.s. planes) are estimated using the full covariance matrix. The cell esds are taken into account individually in the estimation of esds in distances, angles and torsion angles; correlations between esds in cell parameters are only used when they are defined by crystal symmetry. An approximate (isotropic) treatment of cell esds is used for estimating esds involving l.s. planes.
Refinement. Refinement of F^2^ against ALL reflections. The weighted R-factor wR and goodness of fit S are based on F^2^, conventional R-factors R are based on F, with F set to zero for negative F^2^. The threshold expression of F^2^ > 2sigma(F^2^) is used only for calculating R-factors(gt) etc. and is not relevant to the choice of reflections for refinement. R-factors based on F^2^ are statistically about twice as large as those based on F, and R- factors based on ALL data will be even larger.

### Fractional atomic coordinates and isotropic or equivalent isotropic displacement parameters (Å^2^)

**Table:**

	*x*	*y*	*z*	*U*_iso_*/*U*_eq_	
C1	0.22254 (14)	1.32153 (13)	1.02506 (11)	0.0359 (3)	
C2	0.01581 (16)	1.31228 (17)	1.00820 (14)	0.0480 (4)	
H2	−0.0583	1.3530	1.0117	0.058*	
C3	0.01374 (15)	1.18158 (17)	0.97786 (13)	0.0453 (4)	
H3	−0.0615	1.1350	0.9632	0.054*	
C4	0.22027 (13)	1.18857 (13)	0.99072 (11)	0.0352 (3)	
C5	0.32330 (13)	1.00672 (13)	0.93800 (13)	0.0387 (3)	
C6	0.30055 (14)	0.99296 (15)	0.82880 (14)	0.0431 (3)	
H6	0.2838	1.0671	0.7838	0.052*	
C7	0.30274 (15)	0.86655 (15)	0.78589 (14)	0.0436 (3)	
H7	0.2868	0.8545	0.7116	0.052*	
C8	0.32871 (13)	0.75997 (13)	0.85454 (13)	0.0386 (3)	
C9	0.35300 (17)	0.77452 (16)	0.96402 (14)	0.0494 (4)	
H9	0.3712	0.7007	1.0092	0.059*	
C10	0.35021 (17)	0.90062 (16)	1.00675 (15)	0.0495 (4)	
H10	0.3664	0.9128	1.0810	0.059*	
C11	0.24232 (12)	0.55046 (13)	0.79327 (11)	0.0333 (3)	
C12	0.26134 (13)	0.41769 (14)	0.76895 (11)	0.0348 (3)	
C13	0.05687 (15)	0.37541 (16)	0.74872 (13)	0.0447 (4)	
H13	−0.0108	0.3171	0.7325	0.054*	
C14	0.03882 (14)	0.50456 (16)	0.77375 (13)	0.0437 (3)	
H14	−0.0409	0.5318	0.7752	0.052*	
O1	0.32804 (10)	1.13575 (10)	0.98142 (10)	0.0464 (3)	
O2	0.34166 (10)	0.63330 (10)	0.81253 (10)	0.0475 (3)	
Cl1	0.36216 (4)	1.40754 (4)	1.05877 (4)	0.05003 (12)	
Cl2	0.40796 (4)	0.36482 (4)	0.76640 (4)	0.05393 (13)	
N1	0.12198 (14)	1.38253 (13)	1.03283 (11)	0.0445 (3)	
N2	0.11690 (12)	1.11893 (12)	0.96874 (11)	0.0418 (3)	
N3	0.13237 (11)	0.59329 (12)	0.79629 (11)	0.0397 (3)	
N4	0.17015 (13)	0.33116 (12)	0.74704 (11)	0.0430 (3)	

### Atomic displacement parameters (Å^2^)

**Table:**

	*U*^11^	*U*^22^	*U*^33^	*U*^12^	*U*^13^	*U*^23^
C1	0.0523 (8)	0.0232 (6)	0.0317 (7)	0.0015 (6)	0.0104 (6)	−0.0007 (5)
C2	0.0536 (9)	0.0444 (9)	0.0482 (9)	0.0148 (7)	0.0173 (7)	−0.0002 (7)
C3	0.0455 (8)	0.0433 (9)	0.0490 (9)	0.0019 (7)	0.0158 (7)	−0.0011 (7)
C4	0.0440 (7)	0.0246 (6)	0.0371 (7)	0.0019 (5)	0.0111 (6)	−0.0030 (5)
C5	0.0363 (7)	0.0233 (6)	0.0580 (9)	−0.0015 (5)	0.0153 (6)	−0.0111 (6)
C6	0.0485 (8)	0.0272 (7)	0.0546 (9)	−0.0001 (6)	0.0155 (7)	−0.0004 (6)
C7	0.0495 (8)	0.0351 (8)	0.0481 (9)	−0.0036 (6)	0.0163 (7)	−0.0082 (7)
C8	0.0340 (6)	0.0234 (6)	0.0604 (9)	−0.0027 (5)	0.0163 (6)	−0.0116 (6)
C9	0.0643 (10)	0.0274 (7)	0.0568 (10)	0.0043 (7)	0.0171 (8)	0.0004 (7)
C10	0.0648 (10)	0.0356 (8)	0.0478 (9)	0.0022 (7)	0.0149 (8)	−0.0073 (7)
C11	0.0384 (7)	0.0253 (6)	0.0359 (7)	−0.0015 (5)	0.0096 (5)	−0.0044 (5)
C12	0.0435 (7)	0.0268 (7)	0.0332 (7)	0.0020 (5)	0.0088 (6)	−0.0041 (5)
C13	0.0495 (8)	0.0409 (8)	0.0424 (8)	−0.0151 (7)	0.0101 (7)	−0.0062 (6)
C14	0.0374 (7)	0.0441 (8)	0.0486 (9)	−0.0045 (6)	0.0099 (6)	−0.0037 (7)
O1	0.0423 (5)	0.0261 (5)	0.0716 (8)	−0.0031 (4)	0.0168 (5)	−0.0173 (5)
O2	0.0401 (5)	0.0271 (5)	0.0798 (8)	−0.0047 (4)	0.0238 (5)	−0.0206 (5)
Cl1	0.0611 (2)	0.02891 (19)	0.0576 (3)	−0.00820 (16)	0.01178 (19)	−0.00849 (16)
Cl2	0.0508 (2)	0.0412 (2)	0.0695 (3)	0.01017 (16)	0.01584 (19)	−0.01644 (19)
N1	0.0605 (8)	0.0315 (6)	0.0420 (7)	0.0106 (6)	0.0146 (6)	−0.0013 (5)
N2	0.0452 (7)	0.0310 (6)	0.0507 (8)	−0.0008 (5)	0.0154 (6)	−0.0063 (5)
N3	0.0380 (6)	0.0306 (6)	0.0504 (7)	0.0005 (5)	0.0116 (5)	−0.0049 (5)
N4	0.0569 (8)	0.0293 (6)	0.0413 (7)	−0.0071 (5)	0.0107 (6)	−0.0073 (5)

### Geometric parameters (Å, º)

**Table:**

C1—N1	1.2991 (19)	C7—H7	0.9300
C1—C4	1.4047 (19)	C8—C9	1.366 (2)
C1—Cl1	1.7224 (16)	C8—O2	1.4046 (16)
C2—N1	1.335 (2)	C9—C10	1.384 (2)
C2—C3	1.368 (2)	C9—H9	0.9300
C2—H2	0.9300	C10—H10	0.9300
C3—N2	1.3377 (19)	C11—N3	1.3028 (18)
C3—H3	0.9300	C11—O2	1.3486 (16)
C4—N2	1.3065 (19)	C11—C12	1.3987 (19)
C4—O1	1.3415 (17)	C12—N4	1.3048 (19)
C5—C10	1.365 (2)	C12—Cl2	1.7184 (15)
C5—C6	1.364 (2)	C13—N4	1.337 (2)
C5—O1	1.4063 (16)	C13—C14	1.364 (2)
C6—C7	1.387 (2)	C13—H13	0.9300
C6—H6	0.9300	C14—N3	1.3383 (19)
C7—C8	1.367 (2)	C14—H14	0.9300
			
N1—C1—C4	121.98 (14)	C8—C9—C10	119.13 (15)
N1—C1—Cl1	118.38 (11)	C8—C9—H9	120.4
C4—C1—Cl1	119.64 (11)	C10—C9—H9	120.4
N1—C2—C3	121.38 (15)	C5—C10—C9	118.84 (16)
N1—C2—H2	119.3	C5—C10—H10	120.6
C3—C2—H2	119.3	C9—C10—H10	120.6
N2—C3—C2	121.84 (15)	N3—C11—O2	120.97 (12)
N2—C3—H3	119.1	N3—C11—C12	121.34 (13)
C2—C3—H3	119.1	O2—C11—C12	117.68 (12)
N2—C4—O1	121.26 (12)	N4—C12—C11	121.92 (13)
N2—C4—C1	121.15 (13)	N4—C12—Cl2	118.13 (11)
O1—C4—C1	117.60 (13)	C11—C12—Cl2	119.96 (11)
C10—C5—C6	122.27 (13)	N4—C13—C14	121.20 (14)
C10—C5—O1	119.01 (15)	N4—C13—H13	119.4
C6—C5—O1	118.56 (14)	C14—C13—H13	119.4
C5—C6—C7	118.91 (15)	N3—C14—C13	121.99 (14)
C5—C6—H6	120.5	N3—C14—H14	119.0
C7—C6—H6	120.5	C13—C14—H14	119.0
C8—C7—C6	118.93 (15)	C4—O1—C5	117.46 (11)
C8—C7—H7	120.5	C11—O2—C8	117.80 (11)
C6—C7—H7	120.5	C1—N1—C2	116.88 (13)
C9—C8—C7	121.91 (13)	C4—N2—C3	116.72 (13)
C9—C8—O2	118.70 (14)	C11—N3—C14	116.74 (12)
C7—C8—O2	119.15 (14)	C12—N4—C13	116.80 (13)
			
N1—C2—C3—N2	−1.7 (3)	N2—C4—O1—C5	5.5 (2)
N1—C1—C4—N2	−2.5 (2)	C1—C4—O1—C5	−174.53 (13)
Cl1—C1—C4—N2	177.22 (12)	C10—C5—O1—C4	−95.31 (18)
N1—C1—C4—O1	177.57 (14)	C6—C5—O1—C4	89.25 (17)
Cl1—C1—C4—O1	−2.73 (19)	N3—C11—O2—C8	11.1 (2)
C10—C5—C6—C7	1.1 (2)	C12—C11—O2—C8	−169.49 (14)
O1—C5—C6—C7	176.40 (13)	C9—C8—O2—C11	86.67 (18)
C5—C6—C7—C8	−0.6 (2)	C7—C8—O2—C11	−98.82 (17)
C6—C7—C8—C9	−0.3 (2)	C4—C1—N1—C2	1.0 (2)
C6—C7—C8—O2	−174.58 (13)	Cl1—C1—N1—C2	−178.69 (12)
C7—C8—C9—C10	0.6 (2)	C3—C2—N1—C1	1.0 (2)
O2—C8—C9—C10	174.91 (14)	O1—C4—N2—C3	−178.35 (14)
C6—C5—C10—C9	−0.8 (2)	C1—C4—N2—C3	1.7 (2)
O1—C5—C10—C9	−176.08 (15)	C2—C3—N2—C4	0.3 (2)
C8—C9—C10—C5	0.0 (3)	O2—C11—N3—C14	178.56 (14)
N3—C11—C12—N4	0.8 (2)	C12—C11—N3—C14	−0.8 (2)
O2—C11—C12—N4	−178.58 (14)	C13—C14—N3—C11	0.0 (2)
N3—C11—C12—Cl2	−179.68 (11)	C11—C12—N4—C13	0.1 (2)
O2—C11—C12—Cl2	0.94 (19)	Cl2—C12—N4—C13	−179.44 (11)
N4—C13—C14—N3	0.9 (3)	C14—C13—N4—C12	−0.9 (2)

### Hydrogen-bond geometry (Å, º)

**Table:**

*D*—H···*A*	*D*—H	H···*A*	*D*···*A*	*D*—H···*A*
C13—H13···N3^i^	0.93	2.60	3.480 (2)	159

Symmetry code: (i) −*x*, *y*−1/2, −*z*+3/2.
